# Complications of 5-azacytidine: Three cases of severe ischemic colitis in elderly patients with myelodysplastic syndrome

**DOI:** 10.3892/ol.2013.1629

**Published:** 2013-10-15

**Authors:** THOMAS MELCHARDT, LUKAS WEISS, LISA PLEYER, SUSANNE STEINKIRCHNER, JUTTA AUBERGER, GEORG HOPFINGER, RICHARD GREIL, ALEXANDER EGLE

**Affiliations:** Department of Internal Medicine III with Hematology, Medical Oncology, Hemostaseology, Infectious Disease and Rheumatology, Oncological Center, Laboratory for Immunological and Molecular Cancer Research, Paracelsus Medical University of Salzburg, Salzburg A-5020, Austria

**Keywords:** 5-azacytidine, myelodysplastic syndrome, colitis, vascular complications

## Abstract

5-Azacytidine (5-AZA) was the first drug to be approved for the treatment of high-risk myelodysplastic syndrome (MDS). The adverse event profile of this drug appears favorable compared with the conventional intensive chemotherapy that is used for MDS or acute myeloid leukemia. However, uncommon adverse events may have remained undetected in the limited number of patients that have been treated to date. The present study describes three cases/66.8 person-years (4,491 cases/100,000 person-years) of severe ischemic colitis in a single center cohort of 95 patients who were consecutively treated using subcutaneous 5-AZA. The results demonstrated a much higher incidence of colitis compared with the rates in the general population or in patients of greater ages and co-morbidities. The present study investigated whether the combination of anemia and constipation due to the co-medication of 5-HT_3_ receptor antagonists may explain the three cases of ischemic colitis.

## Introduction

Myelodysplastic syndrome (MDS), with high-risk features according to the international prognostic scoring system (IPSS), has a poor prognosis (1). However, allogeneic stem-cell transplantation, which is the only curative option, is not viable for the majority of MDS patients due to their age and co-morbidity. 5-Azacytidine (5-AZA) treatment resulted in a significant benefit for patients with high-risk MDS in two randomized clinical phase III trials (2,3). The most recent trial was published in 2009 by the International Azacytidine High-Risk MDS Survival Study Group and resulted in an OS benefit of 9.5 months (24.5 months OS with 5-AZA vs. 15 months OS with conventional care). Based on the data of the two trials, with 549 patients overall, 5-AZA is now approved by the Food and Drug Administration and the European Medicine Agency. The safety profile of this drug appears favorable compared with the conventional chemotherapy that is used for MDS or acute myeloid leukemia (AML). However, uncommon adverse events may have remained undetected in the limited number of patients that were treated in these clinical trials.

## Materials and methods

The present study describes a relevant incidence of colitis in three patients in a single center cohort of 95 MDS patients who were consecutively treated with subcutaneous 5-AZA in the tertiary cancer center of the Paracelsus Medical University of Salzburg (Salzburg, Austria). These patients are part of the Austrian Azacytidine Registry and the basic characteristics and preliminary results have already been published in part in abstract form (4). Written informed consent was obtained from the patients within the Austrian Azacytidine Registry.

## Results

Between 2007 and 2011, 95 patients were treated with 5-AZA at Department of Internal Medicine III, Paracelsus Medical University of Salzburg. The median age of the cohort was 71 years (range, 42–88 years) and 72% of the patients had a diagnosis of refractory anemia with excessive blasts (RAEB). Three of the 95 patients (3.1%) developed severe colitis following a median of one month (range, one to two months) of 5-AZA treatment, with a histological diagnosis of ischemic colitis. The sigmoid colon was involved in all three cases and none of the patients were administered non-steroidal anti-inflammatory drugs (NSAIDs) prior to the diagnosis of colitis. The median age of the three patients was 81 years (range, 81–82 years) and two of the three patients were male. All three patients had MDS with a high-risk profile, including one case of chronic myelomonocytic leukemia (CMML) and two cases of RAEB, and were treated with a median daily dose of 130 mg 5-AZA (range, 100–150 mg) for seven days (Table I). All the patients had a negative JAK2 status and two had already experienced vascular complications prior to the diagnosis of MDS, including stroke and myocardial infarction. All three patients were administered the 5-HT_3_ receptor antagonist, granisetron, as a supportive treatment prior to 5-AZA application.

## Discussion

Patient 1 was an 81-year-old male with constipation, who developed RAEB II following the diagnosis of low-risk MDS for two years. 5-AZA was administered subcutaneously over seven days in an outpatient setting with routine antiemetic treatment using granisetron at a dose of 2 mg/day. At the end of the third cycle, without any previous major side-effects, the patient was admitted to hospital due to abdominal pain, and severe colitis was diagnosed by endoscopy. The patient’s status deteriorated due to severe peritonitis, and following the diagnosis of colonic perforation, a colostomy was performed. The histological examination revealed ischemic colitis and gangrenous tissue. Stool cultures revealed no bacterial or viral infection. The patient developed renal failure due to post-operative sepsis and succumbed despite dialysis at 47 days post-colostomy.

Patient 2 was diagnosed with RAEB I due to severe pancytopenia with neutrophil counts of <500 cells/μl. Due to the unfavorable IPSS score, 5-AZA treatment was initiated and the patient developed a neutropenic fever following the first day of 5-AZA treatment. Subsequent to administering adequate antibiotic treatment using moxifloxacin, the causal treatment with 5-AZA was continued and the patient developed diarrhea with severe colitis, which was diagnosed by endoscopy. The histological examination of the tissue samples also revealed a pattern of ischemic colitis and the stool cultures were without pathological findings. Following the resolution of the colitis and during the treatment with mesalazin, the 5-AZA treatment was continued due to severe thrombopenia from MDS and a complex situation of platelet-specific alloantibodies with refractoriness to platelet substitution. The further treatment was tolerated without any colitis symptoms using a consequent laxative treatment, and was continued until the 7th cycle of 5-AZA due to the initial delay following the first cycle, but without any improvement in the cytopenia. Due to severe thrombopenia, an off-label use of romiplostim was initiated with dose escalation up to 1,000 μg/week, but was subsequently stopped due to a lack of response. The patient was administered the best supportive care. The patient succumbed 13 months after first diagnosis due to blast crisis and a transformation of the disease into AML.

Patient 3 was diagnosed with CMML with 15% blasts in the bone marrow, and also developed abdominal pain and diarrhea during the second cycle of 5-AZA treatment. Ischemic colitis was diagnosed by endoscopy and histological examination of the tissue samples. Following the resolution of the symptoms and mesalazin treatment, 5-AZA was also continued with supportive laxative treatment, resulting in a sustained improvement of the cytopenias and a normalization of the bone marrow blasts. Following 28 months of treatment with a sustained response, the patient succumbed due to pneumonia.

Vascular complications have not been reported to be associated with 5-AZA treatment in the two previously published trials of MDS (2,3). The incidence of ischemic colitis in the general population is estimated to be between four and 10 cases/100,000 person-years, but this increases with age and co-morbidity. Chronic obstructive pulmonary disease (COPD) and an age of >65 years leads to an increase in the incidence to 373 cases/100,000 person-years (5). By contrast, an estimated incidence of 4,491 cases/100,000 person-years (three cases/66.8 person-years) was observed in the small cohort of elderly high-risk MDS patients in the present study. Compared with the data of the COPD-cohort interpreted as a relevant co-morbidity and as a risk factor for vascular events, the incidence appears much higher in the present study of MDS patients treated with 5-AZA. Furthermore, the affect of age as a risk factor for developing ischemic colitis in the general population may also be observed in the present MDS patients. The three patients that experienced this complication had a median age of 81 years (range, 81–82 years) compared to a median age of 71 years (range, 42–88 years) for the whole MDS cohort (95 patients).

The reason for this relatively high incidence of colitis in the present study cohort is unclear. NSAID-associated colitis may be excluded as none of the patients were administered NSAIDs. Furthermore, the re-exposure to 5-AZA in two patients without an adverse event make a direct toxic effect on the mucosa or endothelial tissue unlikely. The present study investigated whether the combination of anemia and constipation may explain the three cases of ischemic colitis. Anemia may lead to a generalized decrease in mucosal perfusion and scybala caused by constipation, leading to epithelial damage and resulting in colitis. Additionally, immunosuppression due to MDS may support a severe course with inflammatory penetration of the intestinal wall.

The following observations may be used to support our hypothesis: Ischemic colitis is more common in irritable bowel syndrome (IBS) compared with the general population (5), therefore indicating a possible causal relation between constipation and ischemic colitis. Additionally, the 5-HT_3_ receptor antagonist, alosetron, which is used for the symptomatic treatment of IBS, was intermittently withdrawn from the market due to a higher incidence of severe constipation and ischemic colitis. Gastrointestinal events, including constipation, are frequently reported during 5-AZA treatment (3), and 5-AZA is a cytostatic drug that is considered to have moderate emetogenic potential (6). Therefore, all the patients in the present study were administered supportive treatment with daily administration of granisetron as a 5-HT_3_ receptor antagonist prior to the subcutaneous 5-AZA application. Considering these facts, the observed cases of ischemic colitis may in part be the result of an anti-emetogenic supportive treatment. This side-effect has not been reported up to now in the context of the large phase III trials (2,3). This may have contributed to the higher median age of the patients in the present study in comparison with these trials (median age in two trials, 69 years vs. the median age of the patients in the present study with colitis, 81 years) and the previously mentioned supportive treatment with daily 5-HT_3_ receptor antagonists in the present cohort.

Due to the observed high rate of constipation, the routine prescription of concomitant laxative agents was implemented in all the patients who were undergoing 5-AZA treatment. Due to the high rate of ischemic colitis, the antiemetic treatment was switched to metoclopramide to avoid this side-effect in the patients with persisting symptoms during the laxative treatment. However, an increased awareness is warranted for this complication in patients who are treated with 5-AZA. Constipation as a possible serious adverse event of 5-AZA treatment should be clearly addressed in the management of these patients.

## Figures and Tables

**Table I:** Main characteristics of patients with ischemic colitis.

Patient	Gender	Age, years	Disease	5-AZA absolute dosage	Re-exposure	Course of colitis	OS since beginning of 5-AZA, months
1	Male	81	RAEB	130 mg, days 1–7	No	Fatal	4
2	Female	82	RAEB	150 mg, days 1–7	Resolved	Resolved	13
3	Male	81	CMML	100 mg, days 1–7	Resolved	Resolved	28

5-AZA, 5-azacytidine; RAEB, refractory anemia with excessive blasts; CMML, chronic myelomonocytic leukemia; OS, overall survival.
